# Inhibition of mouse alkali burn induced-corneal neovascularization by recombinant adenovirus encoding human vasohibin-1

**Published:** 2010-07-26

**Authors:** Shi-you Zhou, Zhao-lian Xie, Ou Xiao, Xiao-ru Yang, Boon Chin Heng, Yasufumi Sato

**Affiliations:** 1 Zhongshan Ophthalmic Center at Sun Yat-sen University, The State Key Laboratory of Ophthalmology, Guangzhou, China; 2 Department of Ophthalmology, The affiliated hospital of Gannan Medical College, Ganzhou City, Jiangxi Province, China; 3 Division of Pulmonary and Critical Care Medicine, Johns Hopkins Asthma and Allergy Center, Baltimore, MD; 4 School of Materials Science & Engineering, Nanyang Technological University, Singapore; 5 Department of Vascular Biology, Institute of Development, Aging, and Cancer, Tohoku University, Sendai, Japan

## Abstract

**Purpose:**

To evaluate the activity of recombinant adenovirus encoding human vasohibin-1 (Ad-Vasohibin-1) on mouse corneal neovasularization induced by alkali burn.

**Methods:**

For the treatment group, 50 mice each received subconjunctival injection (5 μl) of 10^9^ plaque forming units of replication-defective Ad-Vasohibin-1. Control group mice received the same dosage of blank adenoviral vector (AdNull). Five days after injection, corneal neovascularization (CNV) was induced by placing 2.5 μl of 0.1 M NaOH on the right cornea for 30 s. Subsequently, CNV was observed and photographed every 3 days for a total duration of 9 days after the alkali burn. The percentage of neovascularized area was measured and compared with the AdNull control. The expression of human vasohibin-1 protein was detected by immunohistochemistry and western blotting at 5, 8, and 14 days after injection. The mRNA expression levels of murine vascular endothelial growth factor (*Vegf*), VEGF receptor 1 and 2 (*Vegfr1*, *Vegfr2*), and vasohibin-1 (*Vash1*) were analyzed and compared by real time quantitative reverse-transcription polymerase chain reaction.

**Results:**

The percentage of neovascularized area within the cornea was significantly reduced in mice treated with Ad-Vasohibin-1 compared to mice treated with AdNull at every time point after alkali-induced injury (7.11%±3.91% and 15.48%±1.79% of corneal area in the treatment and control groups, respectively, on day 3; 31.64%±4.71% and 43.93%±6.15% on day 6, and 45.02%±9.98% and 66.24%±7.17% on day 9, all p<0.001). Human vasohibin-1 protein was detected at the injection sites on day 3 after corneal burn and was highly expressed in the central subepithelial stroma and co-localized with neovascularized vessels within the alkali-treated cornea on day 6. On day 9, the peripheral cornea exhibited a similar staining pattern as the central cornea, but a more intense vasohibin-1 immunostaining signal was detected in the deep stroma. Some of the vasohibin-1 stain signal diffused into the frontal and deep stroma of the central cornea and was not co-localized with new vessels. By contrast, in mice injected with AdNull or normal corneas, no vasohibin-1 stain signal was detected within the corneas. Vasohibin-1 protein expression within treated corneas was also further confirmed by western blotting on day 5. Expression appeared to peak by day 8 and was maintained at high levels until day 14. However, Vasohibin-1 protein was not detected in the corneas of normal mice or mice treated with AdNull. Real-time quantitative reverse-transcription polymerase chain reaction analysis showed that expression of *Vegfr2* and endogenous *Vash1* mRNA were significantly decreased in the treatment versus control group (*t_1_*=–2.161, *p_1_*=0.047; *t_2_*=–2.236, *p_2_*=0.041). In contrast, there were no significant differences in *Vegf* and *Vegfr1* mRNA expression levels between the treatment and control groups (p>0.05 for both).

**Conclusions:**

Subconjunctival injection of Ad-Vasohibin-1 significantly reduces corneal neovascularization in alkali-treated mouse corneas. This effect of anti-neovascularization may be related to the downregulation of *Vegfr2* expression.

## Introduction

Corneal neovascularization is a severe debilitating condition that results in the loss of immune privilege of the cornea and in visual impairment. It is characterized by corneal ingrowth of new blood vessels originating from the limbus. The mechanism of corneal neovascularization is thought to be the result of an imbalance between angiogenic and anti-angiogenic factors and the predominance of angiogenic factors. Angiogenic factors implicated in the development of corneal neovascularization include vascular endothelial growth factor (VEGF), basic fibroblast growth factor (bFGF), leptin, angiogenin, prostaglandins, interleukin-2 and −8, and platelet-derived growth factor (PDGF) [1]. Several angiogenesis inhibitors have also been identified in the cornea, such as thrombospondin-1 (TSP-1), pigment epithelium-derived factor (PEDF), prolactin, angiostatin, and endostatin [1].

The regulation of angiogenesis is a complicated process that involves a series of regulatory switches. Several treatment modalities are currently used for ocular neovascular diseases, including surgery [2], laser photocoagulation [3], and medication [4,5]. However, there are many limitations and complications associated with each of these treatment modalities. Controlling angiogenesis by natural and/or synthetic angiogenesis inhibitors might be a promising and alternative strategy. Supplementation of exogenous angiogenesis inhibitors, such as angiostatin [6], endostatin [7], antithrombin [8], thrombospondin [9], and PEDF [10], may provide an alterative way to prevent pathological corneal angiogenesis.

Recently, vasohibin-1 (VASH1) has been identified as a novel endothelium-derived negative feedback regulator of angiogenesis and is specifically expressed in endothelial cells. It has been shown to be upregulated in cultured vascular endothelial cells when stimulated by VEGF and fibroblast growth factor 2 [11,12]. The major physiologic effects of vasohibin-1 include inhibition of endothelial cell migration, proliferation, and tube formation. Vasohibin-1 has been shown to suppress angiogenesis in chick chorioallantoic membrane after subcutaneous implantation of matrigel, and in a tumor xenograft model. Overexpression of vasohibin-1 blocks neovascularization in pathological conditions, such as hypoxia-induced retinal ischemia, and adventitial angiogenesis following vascular injury [13,14]. Previously, Watanabe and colleagues [11] showed that recombinant vasohibin-1 protein selectively inhibited corneal angiogenesis in vivo when implanted with bFGF within a mouse corneal micropocket. We therefore hypothesize that vasohibin-1 gene transfer may be a useful strategy in the prevention of corneal neovasularization and may provide a viable future strategy for suppressing corneal neovascularization under pathological conditions. In this study, we investigated the therapeutic potential of exogenous recombinant human vasohibin-1 in inhibiting corneal neovascularization within alkali-treated mouse corneas.

## Methods

### Construction of adenoviral vector expressing human vasohibin-1

A replication-defective adenoviral vector encoding human vasohibin-1 (Ad-Vasohibin-1) was prepared according to our previous protocol [15]. Briefly, the Adenovirus Vector Expression kit (TaKaRa, Ohtsu, Japan) was used to achieve homologous recombination in vivo between the transfer cassette bearing the vasohibin-1 expression unit and the adenovirus genome as well as the restriction enzyme-digested adenovirus genome tagged with terminal protein in human embryonic kidney (HEK) 293 cells. Plaque-purified adenoviruses were propagated in HEK 293 cells. The viral lysates were purified and concentrated through two cycles of CsCl_2_ step gradients. The reconstructed adenovirus was divided into several aliquots and stored at a titer of 1.0×10^9^ pfu (plaque forming unit)/μl at –80 °C. The adenoviral vector without the encoding gene (AdNull) was used as the negative control.

### Animals

The research protocol was approved by the animal care committee of Zhongshan Ophthalmic Center (Guangzhou, China). The animals were handled and fed in accordance with the Association for Research in Vision and Ophthalmology (ARVO) Statement for the Use of Animals in Ophthalmic and Vision Research. For the alkali-induced corneal neovascularization assay, female BALB/c mice (Guangdong Provincial Center for Animal Experiment, Guangzhou City, Guangdong Province, China) were used. A total of 110 mice, aged 6–8-weeks old, with weight ranging from 18 to 20 g were used for this study. Mice with corneal scars or neovascularization were excluded from this study.

### Subconjunctival injection of adenovirus

One hundred out of a total of 110 mice were randomly assigned to the treatment or control groups. In the treatment group, 50 mice received subconjunctival injection of 5 μl of viral solution containing 10^9^ viral particles of Ad-Vasohibin-1. In the control group, 50 mice received subconjunctival injection of the same amount of AdNull. Only the right eye of each mouse was used for injection. The remaining ten mice were used as normal controls without injection.

### Induction of alkali burn-induced corneal neovascularization

Five days after subconjunctival injection, corneal neovascularization was induced by alkali burn, according to the method devised by Ormerod and collegues with modification [16]. Briefly, after general anesthesia with an intraperitoneal injection of a combination of xylazine hydrochloride (5 mg/kg) and ketamine hydrochloride (35 mg/kg; both purchased from HangZhou Peak Chemical Corp., Zhejiang, China), and topical anesthesia with a drop of 0.5% proparacaine hydrochloride (Alcaine eye drops; Alcon Inc., Fort Worth, TX), filter paper (2.5 mm diameter) was immersed in 2.5 μl 0.1 M NaOH and then placed centrally on the mouse cornea for 30 s. The alkali-treated cornea was then irrigated with 60 ml of normal saline. Subsequently, corneal and limbal epithelia were scraped off with a surgical blade under a microscope. Erythromycin ophthalmic ointment was administered immediately after epithelial denudation.

The area of corneal neovascularization was quantified by photographic documentation every 3 days for a total duration of 9 days. Three mice of each group were sacrificed on days 3, 6, and 9 after corneal injury for immunohistochemistry. Corneas from ten mice of each group were procured on days 5, 8, and 14 after alkali treatment for western blot analysis. On day 9 after alkali treatment, corneas from the remaining mice were procured for real-time quantitative reverse transcription PCR (RT–PCR) analysis.

### Quantification of corneal neovascularization

On days 3, 6, and 9 after alkali-induced corneal injury, murine corneas were examined with a digital camera (ORCA ER; Hamamatsu, Hamamatsu City, Japan) attached to a slit-lamp microscope. The images were captured by ImageNet 2000 software (Topcon Medical Systems Inc., Oakland, NJ). The National Institutes of Health (NIH) Image J1.62 software was subsequently used for quantitative image analysis [17] (developed by Wayne Rasband, NIH, Bethesda, MD). The vascularized area was outlined using the innermost vessel of the limbal arcade as the border. The area of neovascularization was normalized to the entire cornea, and the percentage of corneal neovascularization was calculated. Calculation of CNV (Corneal Neovascularization) percentages was performed in a masked fashion.

### Real-time quantitative reverse-transcription polymerase chain reaction

Nine days after the alkali burn, corneas were dissected and RNA samples were isolated using an RNeasy kit (QIAGEN, Valencia, CA). After quantification of the RNA concentration, total RNA was treated with DNase I (Ambion, Austin, TX) to remove any contaminated genomic DNA. One microgram of murine total RNA per 20 μl of reaction volume was reverse transcribed into cDNA using the SuperScript III First-Strand Synthesis System (Invitrogen, Carlsbad, CA). Minus RT (diethylpyrocarbonate [DEPC]-treated water instead of transcriptase when making cDNA) and nontemplate control samples were made at the same time as controls for quantitative real-time PCR amplification. Samples of synthesized cDNA were divided into aliquots and stored at –80 °C.

Real-time quantitative PCR was performed and analyzed using an ABI PRISM 7000 sequence detection system (Applied Biosystems Inc., Foster City, CA). The relative quantitative method was used to determine gene expression levels, as described in detail by Bookout et al. [18,19]. The standard curve method was used for relative quantification of the gene of interest (GOI), such as *Vash1*, *Vegf*, and VEGF receptor 1 and 2 (*Vegfr1* and *Vegfr2*), relative to glyceraldehyde-3-phosphate dehydrogenase (*Gapdh*). Each sample was run in triplicate, and a dilution series of standard cDNA samples was constructed for the GOI and reference gene (*Gapdh*). Reactions were performed in a 20-μl volume using the SYBR Green reaction mix (QIAGEN) with 0.5 mmol/l primers and 2 μl cDNA. The sequences of the PCR primer pairs are listed in Table 1. Thermal cycling consisted of denaturation for 10 min at 95 °C, followed by 40 cycles of 30 s at 95 °C and 1 min at 60 °C.

**Table 1:** Primer sets for real time RT–PCR.

**Gene**	**Forward (5′-3′)**	**Reverse (5′-3′)**
*Vash1*	AGACGCCACTGTCGCTTT	TGTCTTTGGAACTTTGTCTGCAA
*Vegf*	CAGGCTGCTGTAACGATGAA	AATGCTTTCTCCGCTCTGAA
*Vegfr1*	GAGGAGGATGAGGGTGTCTATAGGT	GTGATCAGCTCCAGGTTTGACTT
*Vegfr2*	GCCCTGCCTGTGGTCTCACTAC	CAAAGCATTGCCCATTCGAT
*Gapdh*	CTCATGACCACAGTCCATGC	CACATTGGGGGTAGGAACAC

For analysis, the method of linear regression was applied. The linear regression formula was produced by plotting the Ct (threshold cycle) versus the log nanogram (log ng) of the input standard purified *Gapdh* cDNA. The formulas resulting from the standard curves were used to interpolate the quantities of GOI and reference gene in the unknown samples. For each of the three replicates of a single sample, the average quantity (avg) of target cDNA interpolated from the standard curve, the standard deviation of the average (stdev), and the coefficient of variation (CV) according to the formula CV=stdev/avg were calculated. After removal of outlier points (>17% CV), the avg, stdev, and CV values were recalculated. Only one outlier point per replicate was removed. For each sample, the GOI quantity was normalized to that of the *Gapdh* gene as arbitrary units (AU), according to the following equation: normalized value=avg GOI quantity/avg reference quantity.

### Immunofluorescent staining for human vasohibin-1

For immunofluorescent staining, three eyes of BALB/c mice in each group were enucleated at each of the preset time points, fixed in 10% (volume/volume) neutralized formaldehyde solution, and embedded in paraffin according to established tissue harvesting protocols [11]. Five sections were made in each tissue block. Immunofluorescent staining was performed using a standard indirect fluorescent staining method, as previously described [11]. Briefly, the dewaxed and rehydrated sections were pretreated at 90 °C in a buffer solution (antigen retrieval solution; DAKO, Glostrup, Denmark). Subsequently, the sections were incubated with 8% (v/v) normal donkey serum for 30 min at room temperature to block unspecific binding. After briefly washing, the samples were reacted with rabbit antihuman antibody, which specifically recognizes human vasohibin-1 (dilution factor of 1:300) and with rat antimouse CD31 (anti-PECAM; dilution factor of 1:150; BD PharMingen, San Diego, CA) overnight at 4 °C to assess localization of the endothelium of corneal neovascular vessels. The samples were then washed and incubated with either donkey antirabbit polyclonal Immunoglobulin (IgG) antibody conjugated to Cy3 (1:1,000; Jackson ImmunoResearch Laboratories, West Grove, PA) or donkey antirat IgG conjugated with Fluorescein Isothiocyanate (FITC) for 1 h at room temperature in the dark. Subsequently after washing with PBS (pH 7.4, 137 mM NaCl, 2.7 mM KCl, 4.3 mM Na_2_HPO_4_, 1.47 mM KH_2_PO_4_), the samples were counterstained with Hoechst (1:1,000; Sigma-Aldrich Corp., St. Louis, MO) and viewed under a Nikon fluorescence microscope. For controls, primary antibody was eliminated from the staining procedure.

### Western blot analysis for human vasohibin-1 expression

The expression levels of human vasohibin-1 protein in mouse corneas were evaluated by western blot analysis. Ten corneal samples were procured from each group on days 5, 8, and 14 after subconjunctival injection and immediately stored in liquid nitrogen. Corneal samples were homogenized with a pre-cooled mortar and pestle, pooled together, and placed in lysis buffer containing 50 mmol/l Tris·HCl (pH 7.4), 150 mmol/l NaCl, 1% deoxycholic acid, 0.1% sodium dodecyl sulfate (SDS), and 0.5% nonyl phenoxylpolyethoxylethanol (NP-40), with Complete Protease Inhibitor Cocktail (Cat No. 11697498001; Roche, Mannheim, Germany). Protein concentrations were determined by the micro-bicinchoninic acid (BCA) assay (Cat No. 23235; Pierce Inc., Rockford, IL), following the manufacturer’s instructions. Twenty micrograms of protein were loaded in wells of a 10% SDS gel. After electrophoresis, separated proteins were transferred to a nitrocellulose membrane. The membrane was then incubated in 0.05 mol/l Tris-buffered saline, pH 7.6 (TBS) containing 5% (weight/volume) skimmed milk to block nonspecific binding and then incubated in TBS containing 0.05% Tween-20, 2.5% skim milk, and 0.5 μg/ml mouse antihuman vasohibin-1 monoclonal antibody. After three washes with TBS–Tween-20, the membrane was incubated in horseradish peroxidase-conjugated rabbit antimouse polyclonal antibody (1:5,000; Amersham-Pharmacia Biotech, Piscataway, NJ). For signal detection, Enhanced Chemoluminescence-Plus western blotting detection reagent (Amersham-Pharmacia Biotech) was used. Membranes were exposed to Kodak Biomax films (Kodak Inc., Rochester, NY).

### Statistical analysis

Statistical analysis was performed with the SPSS software for Microsoft Windows (version 17.0; SPSS Inc., Chicago, IL). Values were reported as mean±SD (standard deviation). Each eye was treated as an independent event for statistical analysis of corneal vessels. One-way ANOVA (ANOVA) and the Student’s *t-*test for independent samples were used for assessing differences in the percentages of CNV and relative levels of gene expression (normalized values) between the treatment and control groups at the same time point. A p value <0.05 was considered statistically significant.

## Results

### Reduction of neovascularization within alkali-treated mice corneas after subconjunctival injection of recombinant adenovirus encoding *VASH1*

**Figure 1:**
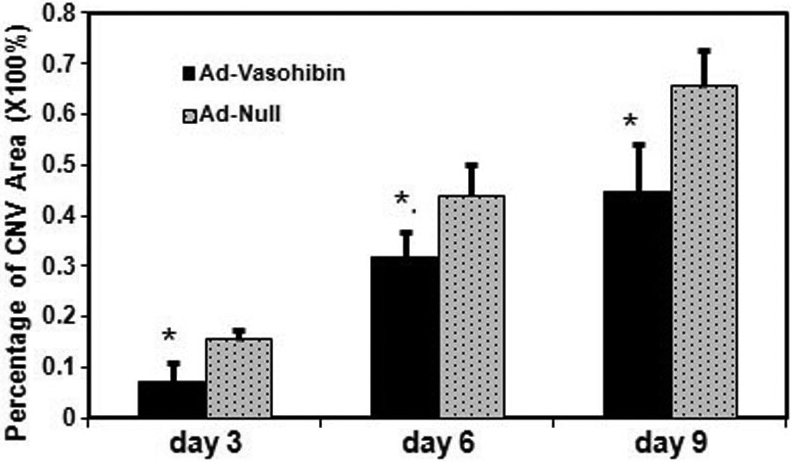
The percentages of neovascularized cornea at each time point. The percentage of neovascularized cornea increased over time in both groups but was significantly reduced in mice with subconjunctival injection of recombinant adenovirus encoding human Vasohibin-1 gene (Ad-Vasohibin-1) compared with the blank adenoviral vector (AdNull) at all time points after alkali-induced injury. On day 3, there was statistical difference in the percentage of CNV area between Ad-Vasohibin-1 (n=38) and Ad Null (n=37) groups (*F*=50.26, *t*=–11.940, 95% CI of the difference: –9.78% to –6.80%). The same results were obtained on day 6 between Ad-Vasohibin-1 (n=25) and Ad Null (n=24) groups (*F*=3.953, *t*=–7.868, 95% CI of the difference: –15.42% to –9.14%) and on day 9 between Ad-Vasohibin-1 (n=22) and Ad Null (n=21) groups (*F*=2.318, *t*=–7.975, 95% CI of the difference: –26.60% to –15.85%). The asterisk indicates a p<0.01.

**Figure 2:**
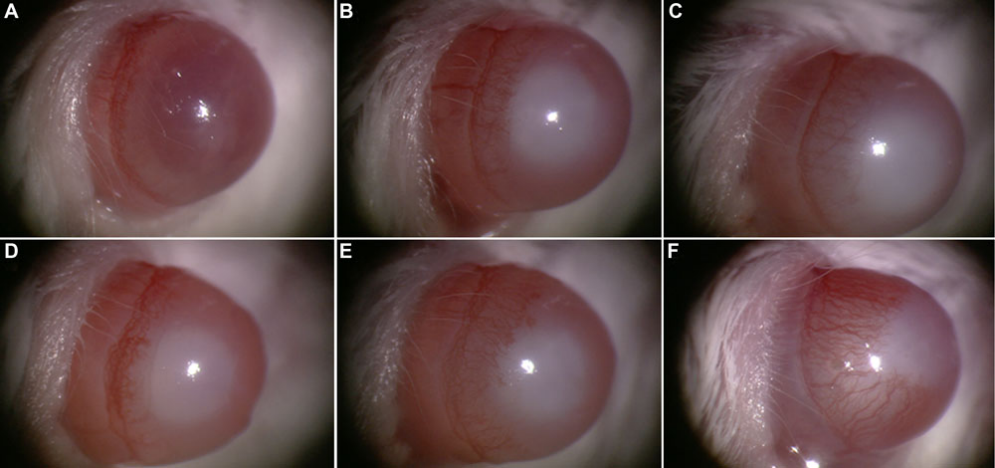
Corneal neovascularization in alkali-treated mice. The area of neovascularized cornea increased over time in both the experimental and control groups but was significantly reduced in mice treated with subconjunctival injection of Ad-Vasohibin-1 (**A**–**C**) compared to the control group with AdNull (**D**–**F**) on day 3 (**A**, **D**), day 6 (**B**, **E**), and day 9 (**C**, **F**) after alkali-induced injury.

The vascularized area after the alkali burn was quantified to evaluate the effect of Ad-Vasohibin-1 on corneal neovascularization. During the experiment, two mice in the treatment group and three mice in the control group died from an overdose of anesthesia before the first time point. The percentages of neovascularized cornea (area) from the treatment and control groups were 7.11%±3.91% (n=38) and 15.48%±1.79% (n=37), respectively, on day 3, 31.64%±4.71% (n=25) and 43.93%±6.15% (n=24), respectively, on day 6, and 45.02%±9.98% (n=22) and 66.24%±7.17% (n=21), respectively, on day 9. Hence, the percentages of neovascularized cornea increased over time in both groups but was significantly reduced in mice with subconjunctival injection of Ad-Vasohibin-1 compared to the AdNull control at every time point after alkali-induced injury (all p<0.001; Figure 1). Corneal neovascularization was therefore significantly reduced by treatment with subconjunctival injection of Ad-Vasohibin-1 (Figure 2).

### Expression of exogenous human vasohibin-1 within alkali-treated mice corneas after subconjunctival injection

**Figure 3:**
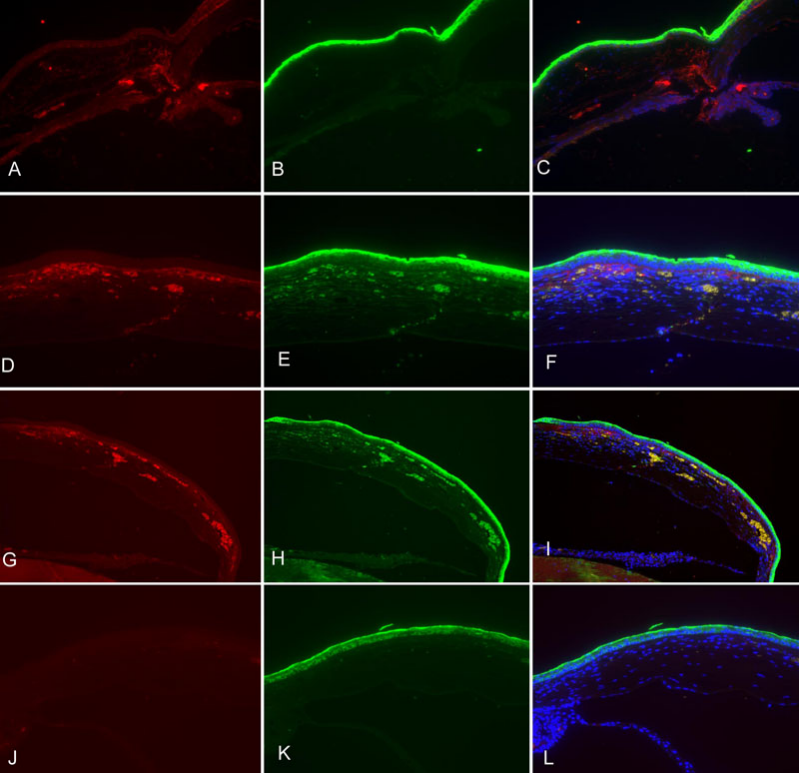
The expression of exogenous human vasohibin-1 protein in mouse corneas at different time points after alkali-induced injury. Ad-Vasohibin-1 was injected subconjunctivally 5 days before alkali-induced corneal injury at a titer of 10^9^ viral particles. **A**–**C**: On day 3 after alkali-induced injury (8 days after injection), vasohibin-1 was expressed within the injection sites and some staining signals were detected at the roots of the iris. **D*–*F**:On day 6 after alkali treatment, vasohibin-1 was highly expressed in the subepithelial stroma of the cornea, and some immunostaining was detected in the deep stroma of the central cornea. Vasohibin-1 expression was highly co-localized with corneal neovascularzation. Immunostaining was diffusely distributed in the frontal stroma, which was not co-localized with new blood vessels within the cornea. **G**–**I**: On day 9, peripheral cornea showed a similar staining pattern as central cornea, but more vasohibin-1 expression was detected in the deep corneal stroma. **J**–**L**: There was no positive immunostaining for vasohibin-1 and CD31 antigen in normal cornea without subconjunctival injection.

The expression of human vasohibin-1 protein was analyzed by fluorescent immunostaining of excised corneas from both the treatment and control groups. We used a specific antibody that recognizes human vasohibin-1 protein, as well as rat antimouse CD31 antibody to detect the endothelial cells of neovascularized vessels. On day 3 after alkali-induced injury, vasohibin-1 was detected at the injection sites. On day 6, it was highly expressed within the central subepithelial stroma and was co-localized with neovascularized vessels in the cornea. On day 9, a similar staining pattern to that observed in the central cornea was displayed by the peripheral cornea, but a more intense vasohibin-1 immunostaining signal was observed in the deep stroma. Some of the vasohibin-1 protein diffused into the frontal and deep stroma of the central cornea and was not co-localized with new blood vessels of the cornea. In contrast, the corneas of normal mice without injection of Ad-Vasohibin-1 and corneas with injection of AdNull displayed negative immunostaining for vasohibin-1 (Figure 3).

**Figure 4:**
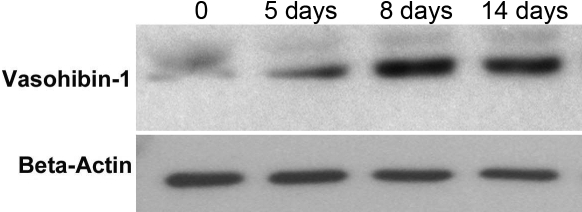
Expression of vasohibin-1 protein within mouse cornea, as detected by western blot analysis. Mice were administered with subconjunctival injection of Ad-Vasohibin-1 at 10^9^ viral particles. Corneas were harvested at different time points, and protein samples were extracted. Ten corneal samples were pooled together for each western blot analysis. A mouse antihuman vasohibin-1 monoclonal antibody was used for the western blot. No vasohibin-1 western blot signal was detected before Ad-Vasohibin-1 was injected. A relatively weak signal was detected on day 5 and was maintained at high levels until day 14 after injection.

Western blot analysis was then performed to compare the expression of human vasohibin-1 protein in the treated and control mice. As shown in Figure 4, the expression of human vasohibin-1 protein in corneas was initially detected on day 5 (time point of alkali-induced corneal injury), subsequently increased to the peak level on day 8, and was maintained at high levels until day 14 after subconjunctival injection of Ad-Vasohibin-1. However, no expression of the protein was detected in the corneas of normal mice and mice injected with AdNull.

### Effect of exogenous vasohibin-1 on the expression of vascular endothelial growth factor, vascular endothelial growth factor receptor-1, vascular endothelial growth factor receptor-2 and endogenous vasohibin-1 in alkali-treated mice corneas

**Figure 5:**
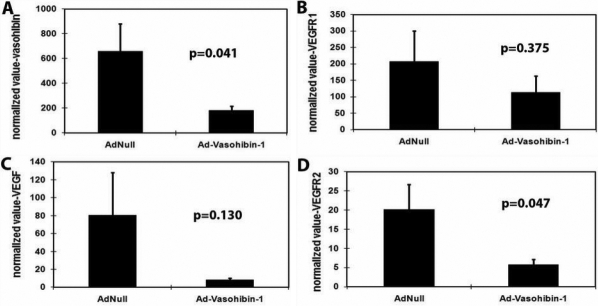
Quantitative comparison of gene expression between the treatment and control groups by real time RT–PCR. The transcript quantification of target genes was normalized relative to the standard housekeeping gene encoding *Gapdh* (arbitrary units, AU). The expression of *Vegfr2* and endogenous *Vash1* mRNA transcripts were significantly decreased in the treatment group compared to the control group (**A**, **B**). Meanwhile, there were slightly decreased but no significant differences in the expression levels of *Vegf* and *Vegfr1* (**C**, **D**) in the treatment versus control group (p>0.05 for both genes).

Real-time quantitative RT–PCR was performed to determine the expression of *Vegf* and its receptors (*Vegfr1*, *Vegfr2*) as well as endogenous *Vash1* between the treatment and control groups (Figure 5). The target genes were normalized relative to the standard housekeeping gene (*Gapdh*). To compare relative expression between groups, all values for quantification of target gene expression were normalized as AU, as described in the Methods section. The mRNA expression level of *Vegfr2* and endogenous *Vash1* significantly decreased in the treatment group (5.80±3.83, 181.08±108.92 AU, respectively) compared to the control group (14.66±11.66, 657.23±630.91 AU, respectively; *Vegfr2*, *F*-ratio=4.672,*t*-value=–2.161,p-value=0.047,95% Confidence Interval: –17.61 to –0.12; *Vash1*, *F*-ratio=4.999, *t*-value=–2.236,p-value=0.041,95% Confidence Interval:–930.07 to –22.23). In contrast, there were no significant differences in the expression levels of *Vegf* between the treated and control mice (8.17±6.28 versus 80.29±135.38, respectively, *F*-ratio=19.77,*t*-value=–1.603,p-value=0.130,95% Confidence Interval: –168.02 to 23.78) and *Vegfr1* (113.85±145.94 versus 206.73±263.29, *F*-ratio=0.844,*t*-value=–0.914,p-value=0.375,95% Confidence Interval:–309.41 to 123.66; p>0.05 for both genes).

## Discussion

Corneal neovascularization alters visual acuity and is induced by inflammatory, infectious, ischemic, degenerative, or traumatic diseases of the cornea and loss of the limbal stem cell barrier. This major ocular complication also results in loss of the immune-privileged status of the cornea, thereby worsening the prognosis of subsequent penetrating keratoplasty. Several clinical studies have shown that corneal neovascularization is the main risk factor of corneal transplant rejection [20], and for highly neovascularized cornea, the rejection rate has been reported as high as 50%. Angiogenesis is an extremely complicate process that is related to vascular endothelial cells. It is a prerequisite that vascular endothelial cells proliferate and migrate to form capillary tubes during angiogenesis. Hence, many therapeutic strategies for anti-angiogenesis involve blocking or inhibiting these processes. Several angiogenesis inhibitors have been investigated for medical treatment, including prolactin [21], somatostatin [22], and thalidomide [23]. Recently, targeted angiostatin and endostatin gene transfer [24,25] has been attempted as a means to halt angiogenesis in a mouse tumor model.

As previous studies have shown, overexpression of VEGF, bFGF, PDGF, nitric oxide synthases, angiopoietin, and angiopoietin 2 were detected in alkali-burned corneas as early as 3 days [1]. VEGF is a key mediator of angiogenesis, and anti-VEGF therapies have been demonstrated to strongly suppress corneal neovascularization [26,27]. Even though anti-VEGF single chain antibody has shown some promise for the treatment of angiogenic-related diseases, such as age-related macular degeneration and tumor angiogenesis, effective endogenous anti-angiogenic molecules still have superb application prospects because they will not interfere with survival pathways.

In this study, we selected vasohibin-1, which is well known to have an anti-angiogenic effect on vascular endothelial cells in vitro and investigated whether vasohibin-1 gene delivery can inhibit corneal neovascularization in an in vivo model. Our results demonstrate that transfer of recombinant adenovirus encoding vasohibin-1 obviously suppressed corneal neovascularization in vivo. The inhibition of corneal neovascularization is associated with a robust expression of exogenous human vasohibin-1, which is detected not only in the endothelial cells of newly formed blood vessels but also within the corneal stroma as a soluble protein. This could possibly indicate that endothelial progenitor cell recruitment into the cornea was reduced.

As was reported previously [11-14], vasohibin-1 can act in a negative feedback loop to help suppress neovascularization within an experimental tumor transplant model, a retinal neovascularization model, and a corneal micropocket model. However, the results of our study showed that the anti-angiogenic effect exhibited by vasoinhibin-1 appears to be VEGF independent and not mediated by suppression of VEGF. The level of *Vegf* mRNA expression was almost the same in both the treatment and control groups. The inhibition of corneal neovascularization was associated with suppression of *Vegfr2* but not *Vegfr1* expression by exogenous vasohibin-1 signaling. This may be achieved by either inhibiting the expression or enhancing the degradation of *Vegfr2* mRNA. The same result was also observed in cultured endothelial cells in vitro [14] and in animal models with retinal neovascularization [13], whereby suppression of *Vegfr2* but not *Vegfr1* reduced the ability of VEGF to induce vasohibin-1. Basic FGF is another major factor implicated in the induction of corneal angiogenesis and has been reported to exert a more potent effect compared to VEGF at the same dosage [28]. FGF function is mediated by binding to its cognate receptor. In a previous study by Sato and colleagues, vasohibin-1 protein inhibited corneal neovascularization induced by bFGF. However, it is unknown whether vasohibin-1 will also have an effect on either the expression or binding of FGF receptors.

Vasohibin inhibits growth, migration, and network formation by endothelial cells. It has two homologs, vasohibin 1 and 2 [29], both of which are expressed in endothelial cells. The expression pattern of vasohibin-2 in vivo resembles that of vasohibin-1 but is generally at a lower level than vasohibin-1 expression and unaffected by stimulation that normally induces vasohibin-1. Vasohibin-2 was previously shown to exhibit anti-angiogenic activity like vasohibin-1 in a mouse corneal micropocket assay when implanted together with bFGF [30]. In this study, only vasohibin-1 cDNA was incorporated within an adenovirus and injected subconjunctivally into the eyes of mice. Vasohibin-1 functions through two isoforms of 42 and 36 kDa that are processed by proteolytic cleavage at a site close to the mutated residues, which include the carboxyl terminal basic domain that possesses activities for heparin binding and anti-angiogenesis [30]. A previous study showed that vasohibin-1 could be a downstream effecter of protein kinase C-delta (PKC-δ) in endothelial cells, which regulate angiogenesis inhibition [31]. In any case, the underlying mechanism of the anti-angiogenic effect of vasohibin-1 has not yet been fully characterized. However, it is unlikely to work antagonistically against VEGF, as shown in this study. Additional studies are needed to elucidate the mechanism by which vasohibin-1 works and find ways to utilize its anti-angiogenic activity for the treatment of corneal diseases.

As shown in the results, endogenous vasohibin-1 was downregulated with lesser corneal neovascularization but was not detected in normal corneas. This implies that the expression of vasohibin-1 is associated with the appearance of corneal neovascularization and suggests that vasohibin-1 may not be an important determining factor of corneal avascularity. The avascularity of the cornea is required for optical clarity and optimal vision. The most crucial factor discovered to date in maintaining corneal avascularity is soluble VEGF receptor-1 [32], which is required to prevent neovascularization. Several other mechanisms may also contribute to corneal avascularity, such as the angiostatic nature of corneal epithelial cells, low levels of pro-angiogenic matrix metalloproteinases (MMPs), several anti-angiogenic factors—including pigment epithelium-derived factor and tissue inhibitor of metalloproteinases—as well as the barrier function of limbal cells [33]. In any case, the mechanisms underlying the maintenance of corneal avascularity remain poorly understood. Currently, no single factor has yet been identified to be critically responsible for maintaining corneal avascularity.

Gene therapy has great potential in treating ocular surface diseases related to cornea because of its accessibility and immune privileged status. Various routes of administration of the target gene have been investigated in experimental studies to test the feasibility of gene transfer to the ocular surface tissues. Intracameral delivery, intrastromal application, topical eyedrops, and subconjunctival injection have all demonstrated encouraging results [34]. However, except for topical and subconjunctival administration, all other gene delivery strategies to the cornea are invasive and result in the damage of corneal integrity. Major determinants for the success of gene therapy are the use of a novel vector and new gene delivery techniques. In our animal model, recombinant replication-defective adenovirus vector was used. Alkali-induced corneal angiogenesis was successfully reduced by subconjunctival injection of Ad-Vasohibin-1. The results thus demonstrated that replication-defective adenovirus vector could provide a highly efficient gene delivery system for ocular surface gene therapy. The recombinant adenovirus was successfully transferred into corneal cells, such as keratocytes, and produced targeted protein. However, the duration of recombinant target gene expression by an adenoviral vector is limited (around 1 month), according to previous studies [11-14]. In our study, a high expression level of vasohibin-1 was observed from day 5 onwards, and expression was maintained at high levels up to day 14 after administration, but expression gradually declined afterwards. As a result, the current approach of using an adenoviral vector may not always be useful for clinical therapy of chronic or slowly progressing degenerative diseases.

Ad-Vasohibin-1 significantly reduced corneal neovascularization in the alkali-treated animal model. We observed that Ad-Vasohibin-1 successfully transduced mouse cornea. A diffused distribution of vasohibin-1 protein expression extending over the cornea was acquired in the alkali-treated mouse cornea. The transgene usually switches on 5 days after adenoviral gene transduction. However, we could not obtain an anti-angiogenic effect when Ad-Vasohibin-1 was injected at the day of corneal alkali burn (data not show). This may be due to the lagging expression of the transferred gene and its lower efficiency of transduction in the damaged keratocytes of burned cornea than in normal cornea before an alkaline burn.

In summary, we demonstrated that topical application of adenovirus encoding human vasohibin-1 has potent inhibitory effects on the progression of corneal neovascularization in vivo. Therefore, vasohibin-1 therapy may be useful as an angiogenic regulator for treating corneal diseases that exhibit neovascularization.
